# 2,2,6-Trimethyl-5-[2-(4-methyl­phen­yl)ethyn­yl]-4*H*-1,3-dioxin-4-one

**DOI:** 10.1107/S1600536809041002

**Published:** 2009-10-17

**Authors:** Ignez Caracelli, Julio Zukerman-Schpector, Adriano S. Vieira, Hélio A. Stefani, Edward R. T. Tiekink

**Affiliations:** a BioMat–Physics Department, Universidade Estadual Paulista Júlio de Mesquita Filho, UNESP, 17033-360 Bauru, SP, Brazil; b Department of Chemistry, Universidade Federal de São Carlos, 13565-905 São Carlos, SP, Brazil; c Departamento de Farmácia, Faculdade de Ciências Farmacêuticas, Universidade de São Paulo, São Paulo-SP, Brazil; d Department of Chemistry, University of Malaya, Kuala Lumpur 50603, Malaysia

## Abstract

The 1,3-dioxin-4-one ring in the title compound, C_16_H_16_O_3_, is in a half-boat conformation with the quaternary O—C(CH_3_)_2_—O atom lying 0.546 (1) Å out of the plane defined by the remaining five atoms. The crystal structure is consolidated by C—H⋯O contacts that lead to supra­molecular layers.

## Related literature

For background to potassium organotrifluoro­borate salts in organic synthesis, see: Caracelli *et al.* (2007 ▶); Stefani *et al.* (2007 ▶); Vieira *et al.* (2008 ▶). For related structures, see: Le & Pagenkopf (2004 ▶); Zukerman-Schpector *et al.* (2009 ▶). For conformational analysis, see: Cremer & Pople (1975 ▶); Iulek & Zukerman-Schpector (1997 ▶).
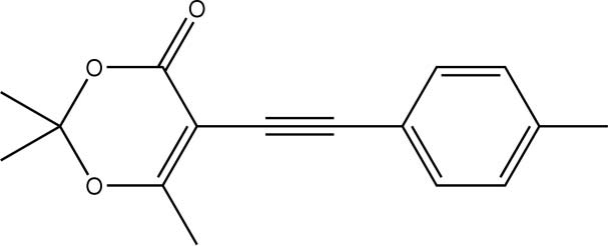


## Experimental

### 

#### Crystal data

C_16_H_16_O_3_
*M* *_r_* = 256.29
Orthorhombic, 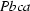

*a* = 14.8486 (15) Å
*b* = 9.621 (1) Å
*c* = 18.9438 (18) Å
*V* = 2706.3 (5) Å^3^
*Z* = 8
Mo *K*α radiation
μ = 0.09 mm^−1^
*T* = 100 K
0.20 × 0.10 × 0.05 mm

#### Data collection

Bruker SMART APEXII diffractometer
Absorption correction: multi-scan (*SADABS*; Sheldrick, 1996 ▶) *T*_min_ = 0.911, *T*_max_ = 1
32445 measured reflections
2382 independent reflections
1727 reflections with *I* > 2σ(*I*)
*R*_int_ = 0.083

#### Refinement

*R*[*F* ^2^ > 2σ(*F* ^2^)] = 0.038
*wR*(*F* ^2^) = 0.096
*S* = 1.04
2382 reflections
174 parameters
H-atom parameters constrained
Δρ_max_ = 0.19 e Å^−3^
Δρ_min_ = −0.17 e Å^−3^

### 

Data collection: *APEX2* (Bruker, 2009 ▶); cell refinement: *SAINT* (Bruker, 2009 ▶); data reduction: *SAINT*; program(s) used to solve structure: *SIR97* (Altomare *et al.*, 1999 ▶); program(s) used to refine structure: *SHELXL97* (Sheldrick, 2008 ▶); molecular graphics: *ORTEP-3* (Farrugia, 1997 ▶) and *DIAMOND* (Brandenburg, 2006 ▶); software used to prepare material for publication: *WinGX* (Farrugia, 1999 ▶).

## Supplementary Material

Crystal structure: contains datablocks global, I. DOI: 10.1107/S1600536809041002/hg2577sup1.cif

Structure factors: contains datablocks I. DOI: 10.1107/S1600536809041002/hg2577Isup2.hkl

Additional supplementary materials:  crystallographic information; 3D view; checkCIF report

## Figures and Tables

**Table 1:** Hydrogen-bond geometry (Å, °)

*D*—H⋯*A*	*D*—H	H⋯*A*	*D*⋯*A*	*D*—H⋯*A*
C16—H16*A*⋯O1^i^	0.98	2.52	3.363 (2)	144
C7—H7⋯O1^ii^	0.95	2.41	3.344 (2)	169

Symmetry codes: (i) 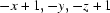; (ii) 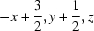.

**Fig. 1.**
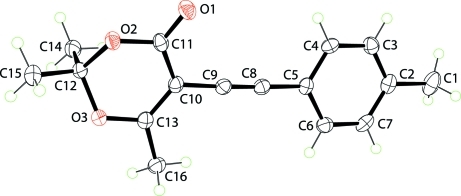
The molecular structure of (I) showing atom labelling scheme and displacement ellipsoids at the 50% probability level (arbitrary spheres for the H atoms).

**Fig. 2.**
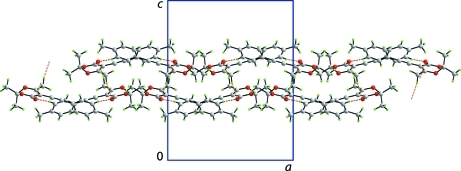
Supramolecular association via C—H···O contacts (orange dashed lines) leading to a layer architecture in the structure of (I).

## supplementary crystallographic information

### Comment

The potential use of potassium organotrifluoroborate salts as intermediates in organic synthesis motivates continuing interest (Caracelli *et al.*, 2007; Stefani *et al.*, 2007; Vieira *et al.* 2008). As a part of on-going studies, the crystal structure of the title compound, (I), is reported herein, which was obtained by the Suzuki-Miyaura palladium-catalyzed cross-coupling reaction of 5-iodo-1,3-dioxin-4-one and a potassium alkynyltrifluoroborate salt.

The molecular structure of (I), Fig. 1, shows the 1,3-dioxin-4-one ring to adopt a half-boat conformation with the C12 atom being displaced 0.546 (1) Å out of the plane defined by the remaining five atoms. The ring-puckering parameters are q_2_ = 0.346 (2) Å, q_3_ = 0.182 (2) Å, Q = 0.391 (2) Å, and φ_2_ = 299.3 (3)° (Cremer & Pople, 1975; Iulek & Zukerman-Schpector, 1997). A similar conformation has been observed in related structures containing the 1,3-dioxin-4-one ring (Le & Pagenkopf, 2004; Zukerman-Schpector *et al.*, 2009). The presence of C—H···O contacts involving the bifurcated carbonyl-O1 atom interacting with two different molecules leads to a layer architecture in the *ab* plane, Fig. 2. These stack along the c direction to form the crystal structure.

### Experimental

The treatment of potassium *p*-tolylethynyltrifluoroborate (244 mg, 1.1 equiv) with K_2_CO_3_ (2 mmol, 276 mg) in 3 ml of degassed THF water (2:1) under an inert atmosphere, followed by the addition of PdCl_2_ (3.5 mg, 2.0 mol%) and 2,2,6-trimethyl-5-iodo-1,3-dioxin-4-one 1 (1.0 equiv) with vigorous stirring for 3 h at 353 K, afforded compound (I) in 72% yield after column chromatography. Single crystals were obtained by slow evaporation from ethyl acetate.

### Refinement

The H atoms were positioned with idealized geometry using a riding model with C—H = 0.95–0.98 Å, and with *U*_iso_ set to 1.2 times (1.5 for methyl) *U*_eq_(parent atom).

### Crystal data

**Table:**

C_16_H_16_O_3_	*F*(000) = 1088
*M**_r_* = 256.29	*D*_x_ = 1.258 Mg m^−^^3^
Orthorhombic, *P**b**c**a*	Mo *K*α radiation, λ = 0.71073 Å
Hall symbol: -P 2ac 2ab	Cell parameters from 4513 reflections
*a* = 14.8486 (15) Å	θ = 2.7–23.7°
*b* = 9.621 (1) Å	µ = 0.09 mm^−^^1^
*c* = 18.9438 (18) Å	*T* = 100 K
*V* = 2706.3 (5) Å^3^	Plate, colourless
*Z* = 8	0.20 × 0.10 × 0.05 mm

### Data collection

**Table:**

Bruker SMART APEXII diffractometer	2382 independent reflections
Radiation source: sealed tube	1727 reflections with *I* > 2σ(*I*)
graphite	*R*_int_ = 0.083
φ and ω scans	θ_max_ = 25.0°, θ_min_ = 2.2°
Absorption correction: multi-scan (*SADABS*; Sheldrick, 1996)	*h* = −17→17
*T*_min_ = 0.911, *T*_max_ = 1	*k* = −8→11
32445 measured reflections	*l* = −22→22

### Refinement

**Table:**

Refinement on *F*^2^	Primary atom site location: structure-invariant direct methods
Least-squares matrix: full	Secondary atom site location: difference Fourier map
*R*[*F*^2^ > 2σ(*F*^2^)] = 0.038	Hydrogen site location: inferred from neighbouring sites
*wR*(*F*^2^) = 0.096	H-atom parameters constrained
*S* = 1.04	*w* = 1/[σ^2^(*F*_o_^2^) + (0.0366*P*)^2^ + 1.3345*P*] where *P* = (*F*_o_^2^ + 2*F*_c_^2^)/3
2382 reflections	(Δ/σ)_max_ < 0.001
174 parameters	Δρ_max_ = 0.19 e Å^−^^3^
0 restraints	Δρ_min_ = −0.17 e Å^−^^3^

### Special details

**Table:**

Geometry. All s.u.'s (except the s.u. in the dihedral angle between two l.s. planes) are estimated using the full covariance matrix. The cell s.u.'s are taken into account individually in the estimation of s.u.'s in distances, angles and torsion angles; correlations between s.u.'s in cell parameters are only used when they are defined by crystal symmetry. An approximate (isotropic) treatment of cell s.u.'s is used for estimating s.u.'s involving l.s. planes.
Refinement. Refinement of *F*^2^ against ALL reflections. The weighted *R*-factor *wR* and goodness of fit *S* are based on *F*^2^, conventional *R*-factors *R* are based on *F*, with *F* set to zero for negative *F*^2^. The threshold expression of *F*^2^ > σ(*F*^2^) is used only for calculating *R*-factors(gt) *etc*. and is not relevant to the choice of reflections for refinement. *R*-factors based on *F*^2^ are statistically about twice as large as those based on *F*, and *R*- factors based on ALL data will be even larger.

### Fractional atomic coordinates and isotropic or equivalent isotropic displacement parameters (Å^2^)

**Table:**

	*x*	*y*	*z*	*U*_iso_*/*U*_eq_	
O1	0.44450 (8)	−0.15682 (13)	0.61722 (7)	0.0282 (3)	
O2	0.33370 (8)	−0.01835 (13)	0.58506 (6)	0.0238 (3)	
O3	0.36181 (8)	0.21026 (13)	0.54478 (6)	0.0225 (3)	
C1	1.01927 (13)	−0.0304 (3)	0.71504 (11)	0.0403 (6)	
H1A	1.0539	−0.0704	0.6760	0.060*	
H1B	1.0211	−0.0935	0.7556	0.060*	
H1C	1.0454	0.0594	0.7284	0.060*	
C2	0.92289 (12)	−0.0097 (2)	0.69219 (9)	0.0261 (5)	
C3	0.85526 (12)	−0.0999 (2)	0.71392 (9)	0.0260 (4)	
H3	0.8698	−0.1753	0.7443	0.031*	
C4	0.76733 (12)	−0.08212 (19)	0.69223 (9)	0.0234 (4)	
H4	0.7221	−0.1442	0.7084	0.028*	
C5	0.74413 (12)	0.02656 (19)	0.64657 (8)	0.0208 (4)	
C6	0.81219 (12)	0.1170 (2)	0.62428 (10)	0.0254 (4)	
H6	0.7982	0.1909	0.5929	0.030*	
C7	0.89939 (12)	0.0998 (2)	0.64750 (10)	0.0273 (5)	
H7	0.9444	0.1637	0.6328	0.033*	
C8	0.65282 (13)	0.0444 (2)	0.62380 (9)	0.0230 (4)	
C9	0.57567 (12)	0.06035 (19)	0.60694 (9)	0.0226 (4)	
C10	0.48224 (12)	0.07787 (19)	0.59006 (9)	0.0216 (4)	
C11	0.42172 (12)	−0.0405 (2)	0.60048 (9)	0.0222 (4)	
C12	0.30049 (12)	0.12229 (19)	0.58355 (10)	0.0218 (4)	
C13	0.45014 (12)	0.19506 (19)	0.55944 (9)	0.0213 (4)	
C14	0.28969 (12)	0.1777 (2)	0.65791 (9)	0.0259 (5)	
H14A	0.2478	0.1183	0.6841	0.039*	
H14B	0.2659	0.2727	0.6561	0.039*	
H14C	0.3483	0.1781	0.6816	0.039*	
C15	0.21469 (12)	0.1198 (2)	0.54194 (10)	0.0278 (5)	
H15A	0.1704	0.0612	0.5661	0.042*	
H15B	0.2265	0.0820	0.4948	0.042*	
H15C	0.1910	0.2145	0.5377	0.042*	
C16	0.50431 (13)	0.3156 (2)	0.53576 (9)	0.0258 (4)	
H16A	0.5116	0.3120	0.4844	0.039*	
H16B	0.5636	0.3126	0.5584	0.039*	
H16C	0.4735	0.4019	0.5489	0.039*	

### Atomic displacement parameters (Å^2^)

**Table:**

	*U*^11^	*U*^22^	*U*^33^	*U*^12^	*U*^13^	*U*^23^
O1	0.0285 (7)	0.0213 (7)	0.0350 (8)	0.0050 (6)	−0.0016 (6)	−0.0007 (6)
O2	0.0197 (7)	0.0194 (7)	0.0322 (7)	0.0015 (6)	−0.0015 (6)	−0.0009 (6)
O3	0.0202 (7)	0.0228 (7)	0.0244 (6)	0.0018 (6)	−0.0003 (5)	0.0029 (6)
C1	0.0234 (12)	0.0672 (17)	0.0302 (11)	0.0004 (11)	−0.0027 (9)	−0.0092 (11)
C2	0.0218 (10)	0.0367 (12)	0.0199 (9)	0.0010 (9)	−0.0010 (8)	−0.0103 (8)
C3	0.0266 (11)	0.0284 (11)	0.0229 (9)	0.0036 (9)	−0.0020 (8)	−0.0008 (8)
C4	0.0245 (11)	0.0238 (10)	0.0220 (9)	−0.0055 (9)	0.0021 (8)	−0.0013 (8)
C5	0.0201 (9)	0.0243 (10)	0.0179 (8)	0.0008 (8)	0.0006 (7)	−0.0051 (8)
C6	0.0283 (11)	0.0233 (11)	0.0246 (10)	−0.0004 (9)	0.0033 (8)	0.0001 (8)
C7	0.0231 (10)	0.0316 (11)	0.0271 (10)	−0.0089 (9)	0.0074 (8)	−0.0065 (9)
C8	0.0242 (11)	0.0242 (11)	0.0206 (9)	−0.0004 (8)	0.0019 (8)	−0.0007 (8)
C9	0.0235 (11)	0.0244 (10)	0.0200 (9)	0.0003 (8)	0.0028 (8)	0.0003 (8)
C10	0.0209 (10)	0.0247 (11)	0.0193 (9)	0.0024 (8)	0.0019 (7)	−0.0021 (8)
C11	0.0231 (10)	0.0232 (11)	0.0202 (9)	0.0055 (8)	−0.0008 (7)	−0.0029 (8)
C12	0.0195 (10)	0.0197 (10)	0.0262 (10)	0.0024 (8)	0.0021 (8)	0.0020 (8)
C13	0.0219 (10)	0.0255 (11)	0.0165 (8)	0.0018 (8)	0.0012 (7)	−0.0041 (8)
C14	0.0243 (10)	0.0272 (11)	0.0262 (10)	0.0036 (9)	0.0017 (8)	−0.0001 (8)
C15	0.0222 (10)	0.0287 (11)	0.0325 (10)	0.0020 (9)	−0.0023 (8)	−0.0028 (9)
C16	0.0266 (10)	0.0257 (11)	0.0250 (9)	−0.0006 (9)	0.0034 (8)	0.0004 (8)

### Geometric parameters (Å, °)

**Table:**

O1—C11	1.211 (2)	C6—H6	0.9500
O2—C11	1.356 (2)	C7—H7	0.9500
O2—C12	1.440 (2)	C8—C9	1.199 (2)
O3—C13	1.349 (2)	C9—C10	1.434 (3)
O3—C12	1.444 (2)	C10—C13	1.355 (3)
C1—C2	1.508 (3)	C10—C11	1.464 (3)
C1—H1A	0.9800	C12—C15	1.498 (2)
C1—H1B	0.9800	C12—C14	1.515 (2)
C1—H1C	0.9800	C13—C16	1.481 (3)
C2—C3	1.390 (3)	C14—H14A	0.9800
C2—C7	1.395 (3)	C14—H14B	0.9800
C3—C4	1.379 (2)	C14—H14C	0.9800
C3—H3	0.9500	C15—H15A	0.9800
C4—C5	1.400 (2)	C15—H15B	0.9800
C4—H4	0.9500	C15—H15C	0.9800
C5—C6	1.399 (3)	C16—H16A	0.9800
C5—C8	1.433 (3)	C16—H16B	0.9800
C6—C7	1.378 (3)	C16—H16C	0.9800
			
C11—O2—C12	118.82 (14)	O1—C11—O2	118.10 (17)
C13—O3—C12	116.42 (14)	O1—C11—C10	125.63 (17)
C2—C1—H1A	109.5	O2—C11—C10	116.10 (16)
C2—C1—H1B	109.5	O2—C12—O3	110.17 (13)
H1A—C1—H1B	109.5	O2—C12—C15	106.64 (15)
C2—C1—H1C	109.5	O3—C12—C15	106.15 (14)
H1A—C1—H1C	109.5	O2—C12—C14	110.40 (15)
H1B—C1—H1C	109.5	O3—C12—C14	109.47 (15)
C3—C2—C7	118.06 (17)	C15—C12—C14	113.88 (15)
C3—C2—C1	121.20 (19)	O3—C13—C10	121.37 (17)
C7—C2—C1	120.74 (18)	O3—C13—C16	112.39 (16)
C4—C3—C2	121.19 (18)	C10—C13—C16	126.19 (16)
C4—C3—H3	119.4	C12—C14—H14A	109.5
C2—C3—H3	119.4	C12—C14—H14B	109.5
C3—C4—C5	120.65 (17)	H14A—C14—H14B	109.5
C3—C4—H4	119.7	C12—C14—H14C	109.5
C5—C4—H4	119.7	H14A—C14—H14C	109.5
C6—C5—C4	118.24 (17)	H14B—C14—H14C	109.5
C6—C5—C8	121.20 (17)	C12—C15—H15A	109.5
C4—C5—C8	120.56 (17)	C12—C15—H15B	109.5
C7—C6—C5	120.52 (18)	H15A—C15—H15B	109.5
C7—C6—H6	119.7	C12—C15—H15C	109.5
C5—C6—H6	119.7	H15A—C15—H15C	109.5
C6—C7—C2	121.31 (18)	H15B—C15—H15C	109.5
C6—C7—H7	119.3	C13—C16—H16A	109.5
C2—C7—H7	119.3	C13—C16—H16B	109.5
C9—C8—C5	177.91 (18)	H16A—C16—H16B	109.5
C8—C9—C10	177.35 (19)	C13—C16—H16C	109.5
C13—C10—C9	122.26 (17)	H16A—C16—H16C	109.5
C13—C10—C11	119.30 (16)	H16B—C16—H16C	109.5
C9—C10—C11	118.19 (16)		
			
C7—C2—C3—C4	−0.1 (3)	C13—C10—C11—O2	−6.8 (2)
C1—C2—C3—C4	−179.09 (17)	C9—C10—C11—O2	178.82 (15)
C2—C3—C4—C5	1.1 (3)	C11—O2—C12—O3	45.9 (2)
C3—C4—C5—C6	−0.7 (3)	C11—O2—C12—C15	160.69 (14)
C3—C4—C5—C8	179.67 (17)	C11—O2—C12—C14	−75.12 (19)
C4—C5—C6—C7	−0.7 (3)	C13—O3—C12—O2	−45.2 (2)
C8—C5—C6—C7	178.97 (17)	C13—O3—C12—C15	−160.28 (15)
C5—C6—C7—C2	1.7 (3)	C13—O3—C12—C14	76.39 (18)
C3—C2—C7—C6	−1.2 (3)	C12—O3—C13—C10	20.6 (2)
C1—C2—C7—C6	177.71 (18)	C12—O3—C13—C16	−161.70 (14)
C12—O2—C11—O1	163.63 (15)	C9—C10—C13—O3	−179.15 (15)
C12—O2—C11—C10	−20.8 (2)	C11—C10—C13—O3	6.7 (2)
C13—C10—C11—O1	168.37 (17)	C9—C10—C13—C16	3.5 (3)
C9—C10—C11—O1	−6.0 (3)	C11—C10—C13—C16	−170.62 (16)

### Hydrogen-bond geometry (Å, °)

**Table:**

*D*—H···*A*	*D*—H	H···*A*	*D*···*A*	*D*—H···*A*
C16—H16A···O1^i^	0.98	2.52	3.363 (2)	144
C7—H7···O1^ii^	0.95	2.41	3.344 (2)	169

Symmetry codes: (i) −*x*+1, −*y*, −*z*+1; (ii) −*x*+3/2, *y*+1/2, *z*.
